# Development and Processing of New Composite Materials Based on High-Performance Semicrystalline Polyimide for Fused Filament Fabrication (FFF) and Their Biocompatibility

**DOI:** 10.3390/polym14183803

**Published:** 2022-09-11

**Authors:** Igor Polyakov, Gleb Vaganov, Andrey Didenko, Elena Ivan’kova, Elena Popova, Yuliya Nashchekina, Vladimir Elokhovskiy, Valentin Svetlichnyi, Vladimir Yudin

**Affiliations:** 1 Institute of Biomedical Systems and Biotechnology, Peter the Great St. Petersburg Polytechnic University, Polytechnicheskaya St. 29, St. Petersburg 195251, Russia; 2 Institute of Macromolecular Compounds of Russian Academy of Sciences, V.O., Bol’shoy Pr. 31, St. Petersburg 199004, Russia; 3 Institute of Cytology, Russian Academy of Sciences, St. Petersburg 194064, Russia

**Keywords:** polyimide, FFF-printing, additive technologies, nanocomposites, carbon fiber, biocompatibility

## Abstract

Samples of composite materials based on high-performance semicrystalline polyimide R-BAPB (based on the dianhydride R: 1,3-bis-(3′,4,-dicarboxyphenoxy)benzene and diamine BAPB: 4,4′-bis-(4″-aminophenoxy)diphenyl)) filled with carbon nanofibers and micron-sized discrete carbon fibers were obtained by FFF printing for the first time. The viscosity of melts of the composites based on R-BAPB, thermal, mechanical characteristics of the obtained composite samples, their internal structure, and biocompatibility were studied. Simultaneously with FFF printing, samples were obtained by injection molding. The optimal concentrations of carbon fillers in polyimide R-BAPB for their further use in FFF printing were determined. The effect of the incorporation of carbon fillers on the porosity of the printed samples was investigated. It was shown that the incorporation of carbon nanofibers reduces the porosity of the printed samples, which leads to an increase in deformation at break. Modification of polyimide with discrete carbon fibers increases the strength and Young’s modulus sufficiently but decreases the deformation at break. The cytotoxicity analysis showed that the obtained composite materials are bioinert.

## 1. Introduction

Fused filament fabrication is the most popular approach among the additive technologies [1,2]. The frequency of application of the FFF method can be explained by the low cost of printing equipment, easy maintenance, and, at the same time, the possibility of obtaining high-quality parts of complex configuration. One of the main problems of FFF technology for functional application is the low mechanical properties of the obtained products in comparison with traditional production methods, such as extrusion or injection molding. This is due to the unavoidable presence of pores because of the layered deposition of the material [3,4], insufficient adhesion between the layers, as well as the tendency of thermoplastic polymers to shrink while cooling during printing [5]. To improve the mechanical characteristics of these products, there are two main solutions: use of new materials or modification of existing ones [6,7]. Currently, FFF printing most often uses such polymers as ABS (acrylonitrile butadiene styrene) plastic and polylactide, as well as polyamides with low mechanical characteristics (their strength does not exceed 60 MPa). To increase the mechanical properties of polymer samples processed by FFF, it is necessary to involve high-performance thermoplastic polymers for this type of printing. Materials obtained from high-performance thermoplastics are distinguished not only by high strength characteristics, but also have increased thermal, fire, and frost resistance, high fracture toughness, chemical resistance to aggressive liquids, and some other special properties. One of the representatives of this class of thermoplastics are polyimides. Products from this class of thermoplastics primarily ensure the stable operation of products obtained from them at high temperatures [8]. Polyimides are used in the production of structural materials, coatings in the aerospace industry, and the bases of flexible printed circuit boards, and they are also promising structural materials for biomedical purposes, due to such operational characteristics as heat resistance, chemical stability, hydrophobicity, and bioinertness [9].

**Figure 1:**
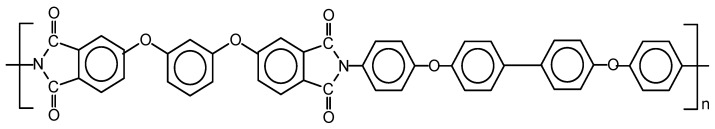
Chemical structure of the polyimide R-BAPB.

One of the promising thermoplastic polyimides is the semicrystalline polyimide R-BAPB based on the dianhydride R: 1,3-bis-(3′,4,-dicarboxyphenoxy)benzene and diamine BAPB: 4,4′-bis-(4″-aminophenoxy)diphenyl) (Figure 1).

The glass transition temperature of R-BAPB is about 205 °C and the melting point is about 318 °C [10]. A main feature of polyimide R-BAPB is the ability to control crystallization and recrystallization at low melt viscosity (up to 1000 Pa∙s at 360 °C), which contributes to its processing by traditional methods suitable for thermoplastics (injection molding, extrusion, hot pressing, etc.). The ability to crystallize from the melt allows one to increase the actual temperature of operation, wear resistance, chemical resistance, and several other operational characteristics of products. To increase the crystallization rate, it is possible to add some nucleants, for example, various carbon nanoparticles [11].

Along with the use of high-performance plastics, an effective way to improve the 3D-printing process and the characteristics of the resulting products is the introduction of various fillers into the polymer material [12,13]. Composite materials are used to achieve the desired mechanical and functional properties by improving the matrix material by adding particles, fibers, or nanomaterials [14,15,16].

Over the last decade, polymer nanocomposites have received much attention, both in fundamental and applied research, due to the possibility of controlling the operational properties of lightweight materials based on them by adding nanofillers. The reason for this is that the nanofillers have a significantly higher ratio of surface area to volume compared to micro- and macrofillers [17]. In particular, the incorporation of carbon nanotubes and nanofibers has a positive effect on the mechanical characteristics of products, increasing the Young’s modulus and tensile strength [18].

The study [19] compared the behavior of amorphous polyetherimide (PEI) (ODPA-P3) with semicrystalline (BPDA-P3) with the incorporation of single-walled carbon nanotubes (SWCNT; 0.1–4.4 vol.%). Tensile tests showed that the yield strength was the same for both polyimides when loading SWCNT up to 0.3 vol.%. Above this concentration, the yield strength for the BPDA-P3 nanocomposites remained constant, whereas for the ODPA-P3 nanocomposites, it increased from 80 up to 126 MPa (1.2 vol.%). In our previous work [20], we showed the influence of carbon nanofibers such as VGCF (vapor grown carbon fiber) on the mechanical properties of polyethyrimide samples processed by FFF. The incorporation of 1 wt.% VGCF into the polyethyrimide increased the strength and Young’s modulus of the printed samples and these values approached those values of the PEI samples processed by injection molding. In [21], it was noted that with an increase in the mass fraction of the carbon nanofibers incorporated into ABS plastic, the tensile strength of FFF-printed samples also increased. Incorporation of 2 wt.% carbon nanofibers into the ABS-matrix resulted in a 40% increase in strength compared to pure ABS. Similarly, an increase of 26.1% in strength was observed for ABS with 1 wt.% carbon nanofibers. The authors in [22] achieved an increase in strength and Young’s modulus for printed ABS samples filled with VGCF by 39% and 60%, respectively, compared with ABS without filler. The article [23] reports that the tensile strength of the printed samples increased almost 3 times with the addition of 7 wt.% of multi-walled carbon nanotubes in ABS.

Mostly, the improvement in strength with the incorporation of nanoparticles into a volume of polymer is explained by the structure organization of the particles in the matrix, and their additional orientation during printing that provides a small reinforcing effect. The increase in the modulus is facilitated by an increase in the stiffness of products as the particles limit the mobility of macromolecular chains. In addition, well-dispersed fillers have more possibilities for physical or chemical bonding with the macromolecular chains thanks to their large surface area and high surface energy. As a result, when cracks begin to form, an effective stress transfer occurs between the polymer and the nanofillers.

If one talks about microfillers, short fibers with a high degree of anisometry are most often used to modify the polymer matrix of materials for FFF [24]. Glass fibers and carbon fibers are most often used to modify the polymer matrix of materials for FFF [25,26]. Carbon fibers are in some way unique among the reinforcing fibers. The share of their use in the production of the composite materials is constantly growing, which is explained by the high level of their properties. Carbon fibers have the largest Young’s modulus among other fibers and exceed all heat-resistant fibers in specific parameters.

The data in [27] showed that printed PEEK samples with reinforced carbon fiber had significantly better strengths than the pure PEEK in the tensile and bending tests. The tensile strength of PEEK with 5 wt.% of carbon fibers (CF) was 101 MPa. The results in [28] suggest that the addition of carbon fiber or glass fiber to PEEK (polyetheretherketone) can significantly increase the tensile and bending strength, but, at the same time, reduce the deformation at break. The fiber content of 5 wt.% increased the tensile strength up to 94 MPa. The study [29] showed that printed samples based on ABS with a carbon fiber content of 5 wt.% had the highest average tensile strength, and the sample with a carbon fiber content of 7.5 wt.% had the highest average value of Young’s modulus. The tensile strength and Young’s modulus of the samples with a carbon fiber content of 5% and 7.5% increased by 22.5% and 30.5%, respectively. At the same time, compared with the sample made of pure plastic, the composite sample with a carbon fiber content of 5 wt.% had greater bending strength, flexural modulus, and fracture toughness. Products made on the basis of high-performance polymers by the FFF can be used in a variety of fields, for example, in the aerospace and automobile industry for the production of durable and lightweight products of complex shape such as air ducts, turbine parts, and aerodynamics elements. Aurora Flight Sciences, specializing in unmanned aerial vehicles (UAVs), in collaboration with Stratasys, created a UAV from ULTEM using 80% FFF-printed parts [30]. Another study reported on nanosatellites for space applications printed from PEEK. Moreover, researchers created nuclear shielding materials. Their study found that FFF-printing of PEEK composites allows the creation of cheaper and lightweight screens that provide greater protection from low-energy gamma rays than from higher-energy ones [31]. However, one of the most relevant areas of application of high-performance polymers in additive technologies is medicine. Currently, stainless steel, titanium, and their alloys are most often used to produce endoprostheses because of their good corrosion resistance, high mechanical properties, and good biocompatibility. However, the obvious hidden dangers are the harmfulness of the released metal ions and the radiopacity of metal alloys in vivo. Another serious problem is the mismatch of the Young’s modulus between the metal and the surrounding bone tissue, which can cause stress screening after surgery, leading to bone resorption [32]. However, these risks can be avoided by replacing metals with strong biocompatible polymers. In recent years, polyetheretherketone (PEEK) being a highly efficient semicrystalline thermoplastic structural polymer has been recognized as a suitable replacement for metal implants, mainly because the elastic modulus of PEEK (3–4 GPa) is much closer to the modulus of human cortical bone (6–30 GPa), which is much lower than the Young’s modulus of titanium and its alloys (more than 100 GPa) [33]. The limiting factor in the use of polymers is still the sufficiently low strength characteristics (not exceeding 110 MPa) compared to metals. In this regard, active research has been conducted in recent years to improve these parameters.

The aim of the study [34] was to compare the cytotoxicity of polyetheretherketone (PEEK) and polyetherketoneketone (PEKK) with conventional materials for dental implants and abutments, namely titanium alloy (Ti-6Al-4V) and tetragonal polycrystal of zirconium dioxide stabilized with yttrium oxide (Y-TZP). According to the analysis of cellular cytotoxicity and expression of proinflammatory cytokine genes, there were no differences between the materials. The PEEK surface, where fibroblast culture showed the best metabolic activity of cells, looks like the more promising material for the implant abutment. In [35], a series of PEEK composites with different carbon fiber (CF) contents (25 wt.% 30 wt.%, 35 wt.%, and 40 wt.%) was successfully obtained by injection molding. Evaluation of mechanical properties showed that the CF-PEEK composites have a higher bending strength, compressive strength, and hardness than pure PEEK, but lower impact strength. The modulus of elasticity of the CF-PEEK composites was much closer to human bones than to metals. In [36], the structural changes of implants fixed on a sintered polyamide skull model was investigated under the influence of mechanical stress in four simplified models. In a simplified model with a quasistatic load, both implants withstood forces exceeding those capable of causing skull fractures.

In one of our previous works [37], the cytotoxicity of samples based on polyetherimide modified with nanofibers such as VGCF was investigated using the MTT test (tetrazolium dye MTT, which is chemically 3-(4,5-dimethylthiazol-2-yl)-2,5-diphenyltetrazolium bromide). The samples made of both pure polyimide and with the addition of 1 wt.% VGCF did not have a negative effect on human fibroblast culture that may indicate their bioinertness. These materials possess good cell adhesion to the surface and have suitable conditions for cell proliferative activity.

Based on the foregoing, the purpose of the present work is to obtain new composite materials for FFF printing based on high-performance semicrystalline polyimide such as R-BAPB modified with nano- and microsized carbon fillers, as well as to study the possibility of using these materials for medical purposes, investigating their cytotoxicity.

## 2. Materials and Methods

A polyimide R-BAPB was synthesized in the form of a powder based on R (1,3-bis(3′,4-dicarboxyphenoxy) dianhydridebenzene), T_m_ ~164 °C (Techhimprom LLC, Yaroslavl, Russia), and diamine BAPB (4,4′-bis(4″-aminophenoxy)biphenyl), T_m_ ~198–199 °C (VWR International, Radnor, PA, USA). Phthalic anhydride, T_m_ ~131–134 °C (Sigma-Aldrich Co., LLC, St. Louis, MO, USA), was used as a chain growth limiter for the polycondensation reaction. Acetic anhydride, benzene, and triethylamine were supplied by Sigma-Aldrich Co., LLC.

The following fillers were used:

- VGCF—carbon nanofibers obtained by gas phase deposition (Pyrograf^®^-III, Cedarville, OH, USA) with an outer diameter of ~100 nm and a length of 20 to 200 µm.
- CF—discrete carbon fibers (Umatex, Moscow, Russia) with a diameter of ~7 µm and a length of ~7 mm.

### 2.1. Preparation of R-BAPB and R-BAPB-Based Composite Materials Modified with Carbon Nanoparticles

Synthesis of R-BAPB was carried out by chemical imidization. A more detailed description of the synthesis is presented in our early works [10]. To obtain the nanocomposite, the required number of VGCF particles was injected into the resulting polyamide acid solution. As a result of synthesis, pure R-BAPB powder was obtained, as well as powders with different concentrations of the carbon nanofibers VGCF—0.5%, 1%, 3%, and 5% (weight percentages). The synthesized polyimide had an average molecular weight of M_w_ ~20,000 Da [38], determined by the light scattering method. Earlier, the structure of polyimide was proven by IR (infrared) spectroscopy [39].

### 2.2. Preparation of the Composite Materials Based on R-BAPB Modified with Micron-Sized Discrete Carbon Fibers

To obtain a composite with discrete carbon fibers, polyimide powder obtained by the method described above was mixed with the carbon fibers in proportions corresponding to the concentrations studied. Before mixing, both powder and fibers were dried in a vacuum thermostat at a temperature of 150 °C for 1 day. Mixing was carried out in a twin-screw microextruder “DSM Xplore MC5” (Xplore, Sittard, The Netherlands) at a temperature of 370 °C and a screw rotation speed of 50 rpm for 10 min to ensure a more uniform dispersion of the fiber in the melt. As a result, strands of the composite material with carbon fiber concentrations of 10%, 20%, and 30% (weight percentages) were obtained.

### 2.3. Granulation of the Obtained Composite Materials

To achieve the most uniform loading of the material for obtaining filaments for FFF printing, all composites were subjected to granulation. Strands were prepared from pure R-BAPB, and the nanocomposites based on them according to the procedure described in paragraph 2.2. After that, all the strands obtained, including composites with the discrete carbon fibers, were crushed in a laboratory mill to granules~1–3 mm long and 1 mm in diameter.

### 2.4. Study of the Viscosity of the Obtained Materials

The viscosity of the polymer melt was studied on the Physica MCR301 rheometric unit (Anton Paar, Graz, Austria) in the CP25-2 cone-plane measuring system (diameter 25 mm, angle 2°, gap between the cone and the plane 0.05 mm) at a temperature of 360 °C. The test was carried out in an oscillating mode in the frequency range from 100 rad/s down to 1 rad/s.

### 2.5. Thermal Analysis of the Samples

Thermal analysis of the samples was carried out using the method of differential scanning calorimetry (DSC) at the “DSC 204 F1 Phoenix” device (NETZSCH, Selb, Germany) in an inert medium (argon), in the temperature range from 25 °C up to 350 °C at a heating rate of 10 K/min. The degree of crystallinity of the R-BAPB samples was estimated from the enthalpy of melting ΔH_m_ calculated earlier for R-BAPB with a degree of crystallinity of 100%, which was equal to 90 J/g [10]. A total of 3 samples were tested for each material (the deviation for each parameter did not exceed 0.5%).

To study the temperature of thermal degradation, thermogravimetric analysis (TGA) was used, using the TG 209 F1 Iris device (NETZSCH, Selb, Germany). The sample was heated in an inert medium (argon), ranging from a temperature of 30 °C to 800 °C at a speed of 10 K/min.

### 2.6. Obtaining Samples by Injection Molding

To study the mechanical properties and internal structure, as well as to determine the optimal concentrations of the fillers for the subsequent production of the filaments, the samples were obtained from the granules of the studied materials by injection molding. Before molding, all pellets were dried in a vacuum thermostat at a temperature of 150 °C for 1 day. Injection molding was carried out using the DSM Xplore MC5 microextruder (Xplore, Sittard, The Netherlands) and the DSM Xplore IM5.5 micro-injector (Xplore, Sittard, The Netherlands). The samples were prepared according to the following parameters:

- Pure R-BAPB: extruder temperature 360 °C, screw speed 50 rpm, cylinder temperature 370 °C, mold form temperature 180 °C, and pressure 16 bar.
- R-BAPB with carbon nanofibers VGCF: extruder temperature 360 °C, screw speed 50 rpm, cylinder temperature 370 °C, mold form temperature 190 °C, and pressure 16 bar.
- R-BAPB with discrete carbon fiber: extruder temperature 370 °C, screw speed 50 rpm, cylinder temperature 380 °C, mold form temperature 190 °C, and pressure 16 bar.

The material cooled in the mold form for about 10 s. As a result, “dog-bone” samples with a width of 4 mm, a thickness of 2 mm, and a length of the working part of 25 mm were obtained by injection molding.

### 2.7. Filaments Production

Based on studies of the viscosity, and thermal and mechanical properties of the prepared materials, optimal concentrations for both types of the fillers were determined. First, the composite granules were dried in a vacuum thermostat at a temperature of 150 °C for 12 h. Next, the granules were loaded into the DSM Xplore MC5 microextruder (Xplore, Sittard, The Netherlands). After that, the filament was extracted from the melt using the coil of the receiving device. The filaments were obtained with the following parameters:

- Pure R-BAPB: screw speed 35 rpm, chamber temperature 360 °C, chamber force 200 N, and coil speed 250.
- R-BAPB with carbon nanofibers: screw speed 35 rpm, chamber temperature 360 °C, chamber force 250 N, and coil speed 250.
- R-BAPB with discrete carbon fiber: screw speed 50 rpm, chamber temperature 370 °C, chamber force 350 N, and coil speed 150.
- As a result, filaments were obtained from pure R-BAPB, R-BAPB with 1 wt.% VGCF, and R-BAPB with 20 wt.% discrete carbon fiber with a diameter of 1.6–1.85 mm.

### 2.8. FFF Printing

FFF printing was performed on an experimental setup for 3D-printing with high-temperature-resistant plastics. Samples were printed in “dog-bone” form with a width of 4 mm, a thickness of 2 mm, and a working length of 25 mm, which corresponds to the samples obtained by injection molding described in paragraph 2.6. The COMPASS-3D software (ASKON, Saint-Petersburg, Russia) was used to create the “dog-bone” model. The Cura v.4.13.0 software (Ultimaker, Utrecht, The Netherlands) was used as a slicer for setting printing parameters.

Samples from different materials were printed with the following parameters:

- Pure R-BAPB: nozzle diameter 0.4 mm, extruder temperature 365 °C, build platform temperature 180 °C, chamber temperature 150 °C, printing speed 50 mm/s, layer thickness 0.1 mm, raster angle ± 45°, and wall thickness 04 mm.
- R-BAPB + 1 wt.% VGCF: nozzle diameter 0.4 mm, extruder temperature 365 °C, build platform temperature 180 °C, chamber temperature 150 °C, printing speed 50 mm/s, layer thickness 0.1 mm, raster angle ± 45°, and wall thickness 0.4 mm.
- R-BAPB + 20 wt.% CF: nozzle diameter 0.4 mm, extruder temperature 380 °C, build platform temperature 180 °C, chamber temperature 150 °C, printing speed 50 mm/s, layer thickness 0.1 mm, raster angle ± 45°, and wall thickness 0.4 mm.

### 2.9. Investigation of the Mechanical Characteristics

The mechanical properties of the samples were studied at the ElectroPuls E1000 (Instron, Norwood, MA, USA). The samples were tested in the form of the “dog-bone”, 4 mm wide and 2 mm thick, and a working part with a length of 25 mm at a speed of 1 mm/min. At least 5 samples from each material were tested to measure the mechanical properties. According to the test results, the Young’s modulus, tensile strength, and deformation at break of each sample were determined.

### 2.10. Investigation of the Internal Structure of the Samples

The porosity of the samples was investigated using the pycnometric method. A sample of the material was placed in a capillary pycnometer and filled with 96% ethanol at 25 °C. Next, the volume occupied by the material was calculated as the difference with the volume of a pycnometer filled with ethanol. The porosity was calculated based on the differences between the theoretical and experimentally determined volume of the material according to the following formula:

(1) $$P=\frac{V_{t}-\frac{m}{\rho }}{V_{t}}\times 100\%$$

where *P* is porosity; *V_t_* is the volume of the test sample determined by the pycnometric method; ρ is the density of the material; *m* is the mass of the sample measured on analytical scales.

Micrographs of the fracture surface of the block samples at various magnifications were obtained using a Supra-55 VP scanning electron microscope (SEM) (Carl Zeiss, Oberkochen, Germany). To obtain a high-quality fracture surface, the sample was broken in liquid nitrogen. The resulting cleavages of the samples were fixed with a special conductive glue on the microscope holders and a thin layer of platinum was sprayed.

### 2.11. Cytotoxicity Study of the Printed Samples

Human osteosarcoma MG63 cell lines were used to study cytotoxicity. The cells were cultured in a CO_2_ incubator at 37 °C in a humidified atmosphere containing air and 5% CO_2_ in an EMEM nutrient medium (Dulbecco’s modified Eagle’s medium; Gibco) containing 1% essential amino acids, 10% (by volume) thermally inactivated fetal bovine serum (FBS; HyClone Marlborough, MA, USA), 1% L-glutamine, 50 U/mL of penicillin, and 50 mcg/mL of streptomycin. For this experiment, the samples in the form of disks with a diameter of 11 mm printed by FFF were filled with 2 mL of complete nutrient medium and incubated for 1 and 3 days. To assess cytotoxicity, cells in the amount of 5.0 × 10^3^ cells/100 mcL/well were plated in 96-well plates and cultivated for 24 h for their attachment. Then, 100 mcL of the medium was added and then incubated for 72 h. At the end of the incubation period, the medium was removed and 50 mcL/well of EMEM medium with MTT (0.1 mg/mL) was introduced. The cells were incubated in a CO_2_ incubator for 2 h at 37 °C. After removal of the supraplastic fluid, formazane crystals formed by metabolically vital cells were dissolved in dimethyl sulfoxide (50 mcL/well) and the optical density was measured at 570 nm on a flatbed spectrophotometer. Ethical Statement: The MG-63 osteosarcoma cell lines were obtained from the Vertebrate Cell Culture Collection (Institute of Cytology RAS, St-Petersburg, Russia).

Cell adhesion to the surface of the samples was studied using a Supra-55 scanning electron microscope (Carl Zeiss, Oberkochen, Germany). Before placing the samples inside the microscope chamber, a thin conductive layer of platinum was sprayed onto their surface. The accelerating voltage was 3–5 kV.

## 3. Results and Discussion

### 3.1. Investigation of the Viscosity of Melts of Composites Based on R-BAPB

**Figure 2:**
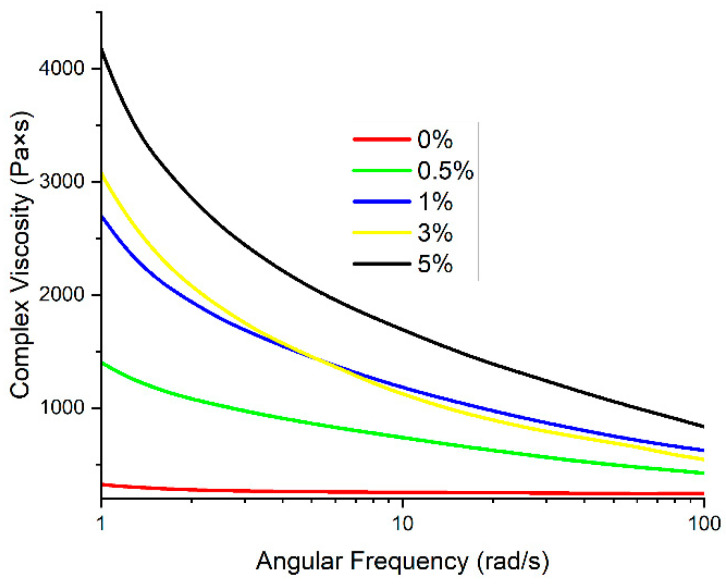
Dependence of the melt viscosity on angular frequency for R-BAPB modified with VGCF.

**Figure 3:**
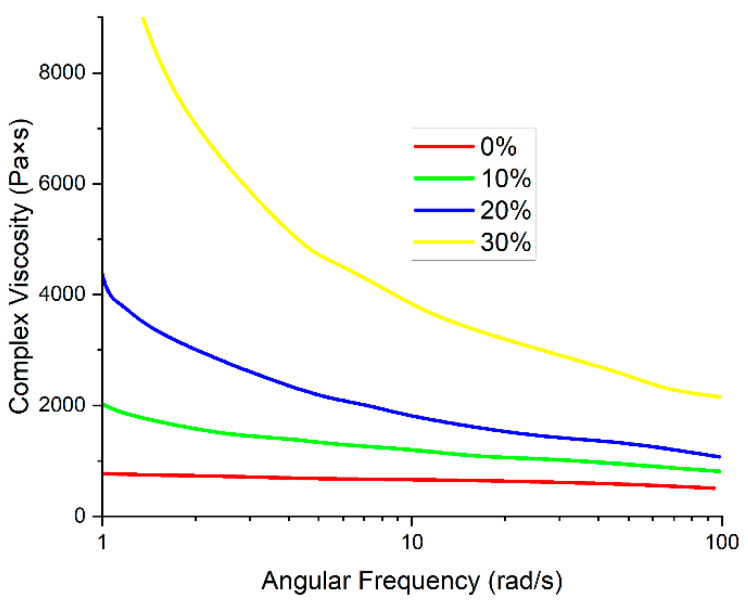
Dependence of melt viscosity on angular frequency for R-BAPB modified with CF.

One of the most important parameters affecting the FFF printing process is the viscosity of the materials. If the viscosity is too high, the movement of the melt may be hindered, and its integrity at the outlet of the nozzle may also be disrupted. The introduction of VGCF particles into the polymer matrix based on R-BAPB leads to an increase in the viscosity with a decrease in angular frequency (Figure 2). The viscosity enhances with an increase in the VGCF concentration. At the angular frequencies of about 1, it increases quite significantly starting from 1 wt.% VGCF, which may be due to the high anisometry of the nanofibers. This fact indicates a good dispersion of the nanoparticles in the polymer matrix [40]. With deformations such as those occurring during the printing process, the melt viscosity is in the range suitable for high-quality printing for all concentrations except 5 wt.% VGCF. The introduction of the discrete carbon fibers leads to a noticeable increase in viscosity (Figure 3). With the introduction of 30 wt.% CF, the melt viscosity reaches 4000 Pa∙s, which is too high for high-quality FFF printing as there is a pure flow and the appearance of breaks in the melt jet. However, for 20 wt.% CF, the value of the complex viscosity at 10 rad/s does not exceed 2000 Pa∙s, which is an acceptable value for processing by both injection molding and FFF printing.

### 3.2. Studies of Thermal Properties of Melts of the R-BAPB-Based Composites

**Figure 4:**
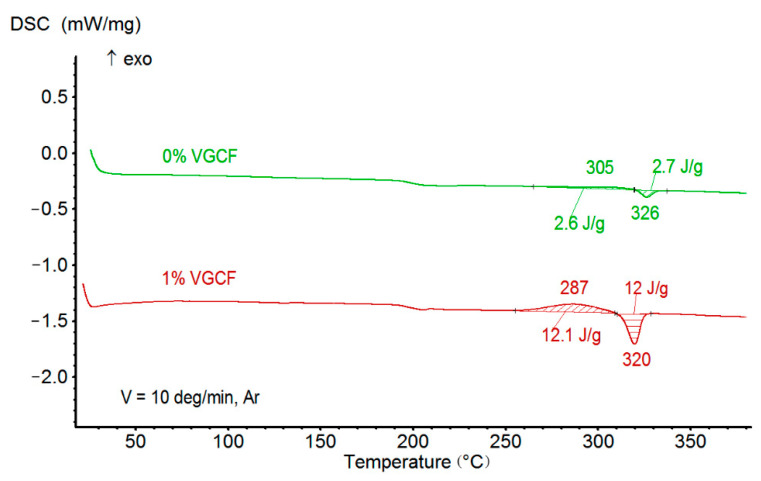
An example of the effect of VGCF on phase transitions in R-BAPB-based composites.

According to the study of materials by the DSC method, the introduction of various concentrations of both VGCF and discrete CF practically does not affect the glass transition temperature (Table 1, Figure 4). At the same time, with an increase in the concentration of VGCF, there is a significant increase in the degree of crystallinity of the nanocomposites and the crystallization rate, which is observed on the DSC curve owing to the appearance of a crystallization peak (Figure 3). All this indicates that the carbon nanofibers act as crystallization centers for polyimide R-BAPB [11]. When the micron carbon fibers are added, a weak peak of crystallization appears during DSC heating. It is worth noting that the samples crystallize under sufficiently slow heating during the DSC experiment. When samples are obtained by injection molding and FFF printing, the composites do not have time to crystallize and remain in an amorphous state.

The temperature of thermal degradation of the materials was determined with the aid of the TGA method. The received data revealed that these materials have a single-stage mechanism of thermal destruction. At the same time, a sharp increase in the rate of the thermal destruction process is observed with the loss of 5% of the sample mass. The addition of different concentrations of the nanofibers has no essential effect on the temperature τ_5_; with the introduction of the discrete carbon fibers, the thermal degradation temperature increases due to the greater thermal stability of the fibers themselves (Table 1).

### 3.3. Investigation of the Mechanical Characteristics and Internal Structure of the Molded and Printed Samples Made of the R-BAPB-Based Composites

**Figure 5:**
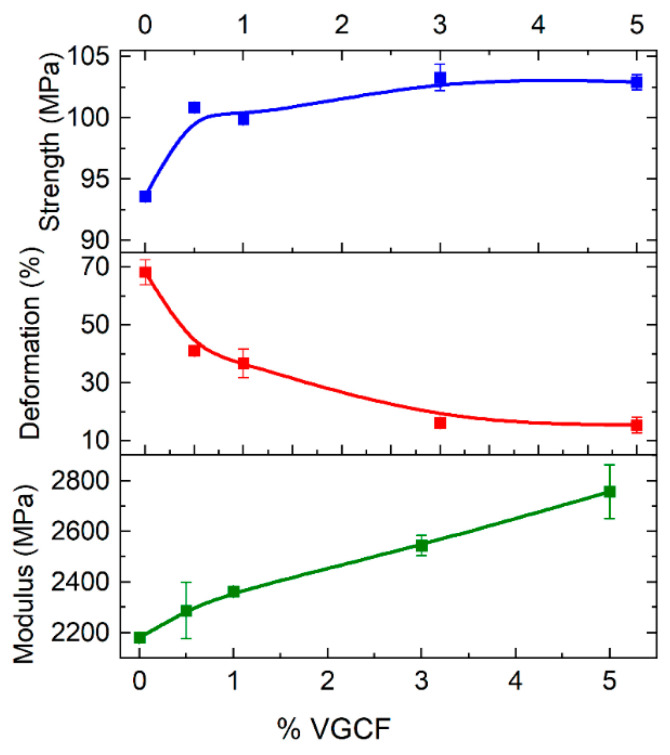
Dependence of strength, Young’s modulus, and deformation at break of the R-BAPB-based molded samples on the concentration of VGCF.

Tensile tests of molded samples were carried out to determine the optimal concentration of the fillers for the preparation of the filament. Analysis of the data showed that with the incorporation of VGCF, the strength and Young’s modulus slightly increase with an increase in VGCF concentration, but the deformation at break significantly decreases (Figure 5). The samples with concentrations of 0.5 wt.% and 1 wt.% VGCF retain about 50% of deformation regarding pure R-BAPB, in contrast to 3 wt.% and 5 wt.%. As the mechanical characteristics of the samples with 0.5 wt.% and 1 wt.% VGCF turned out to be quite close, the choice in favor of 1 wt.% VGCF was made due to a 3 times higher degree of crystallinity (Table 1), because of the presence of a larger amount of crystallization centers. This factor may be important in further studies of the crystallized samples obtained by FFF printing, as this composite will be easier to transfer to a crystallized state due to a higher crystallization rate.

**Figure 6:**
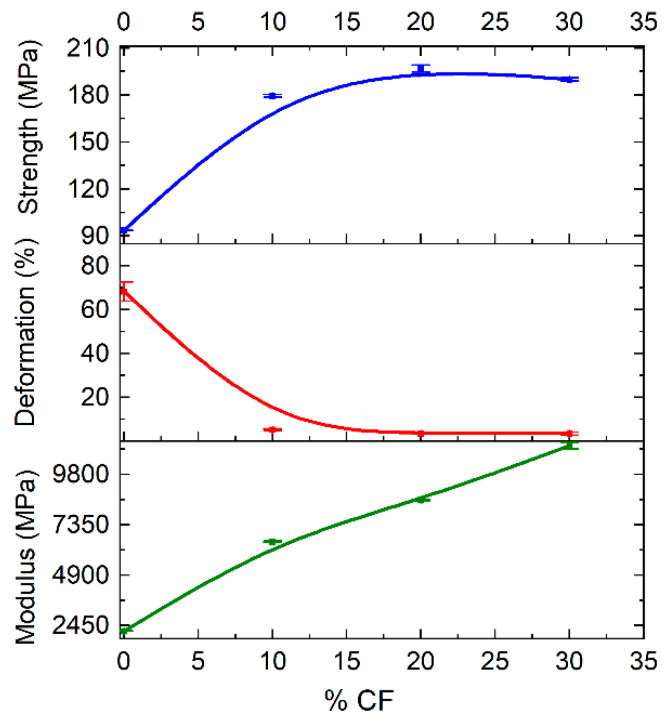
Dependence of strength, modulus of elasticity, and deformation at break of the R-BAPB-based molded samples on the concentration of CF.

The incorporation of the discrete CF leads to a visible increase in the strength and Young’s modulus of the studied samples, while the deformation at break is reduced by more than 10 times starting from 10 wt.% CF (Figure 6). The sample with 30 wt.% CF has a similar strength and a larger modulus than that with 20 wt.%, but due to the high melt viscosity of this composite (Figure 3), a choice was made in favor of R-BAPB with 20 wt.% of CF.

**Figure 7:**
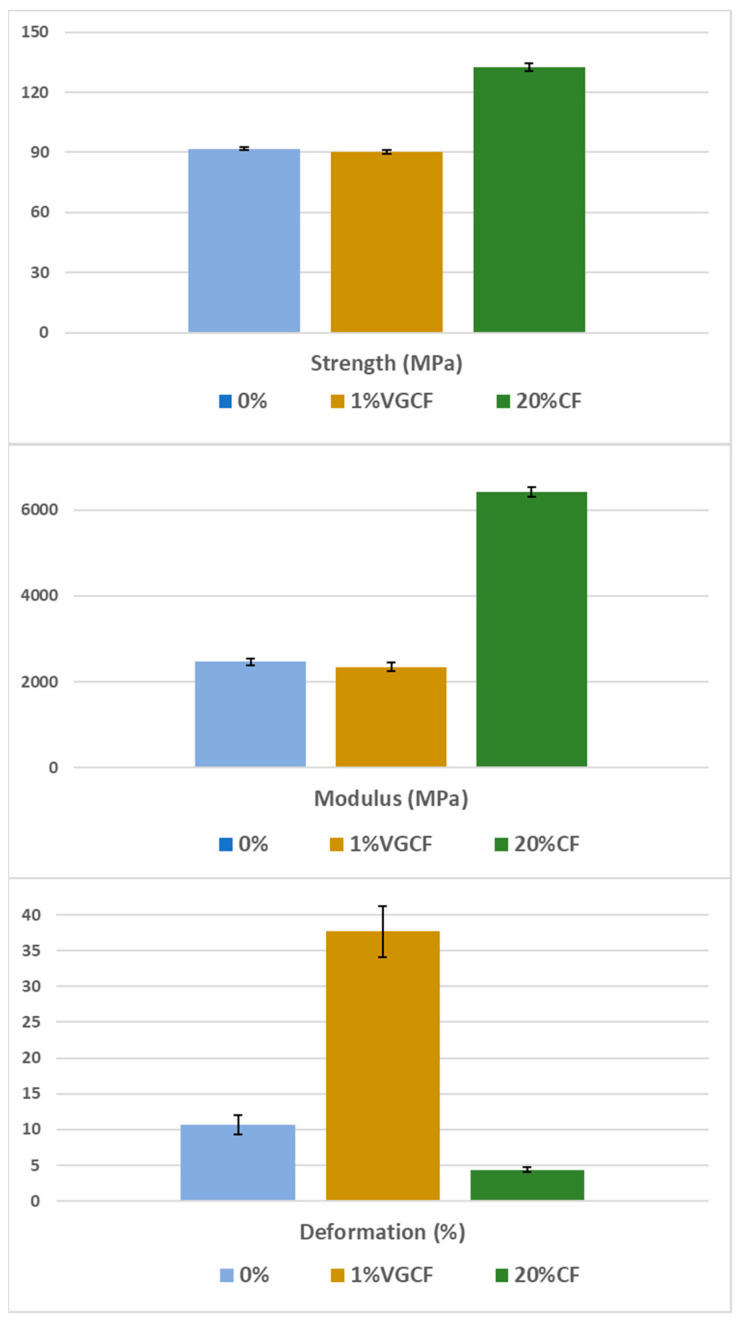
Mechanical characteristics of the FFF samples from R-BAPB and its composites.

**Figure 8:**
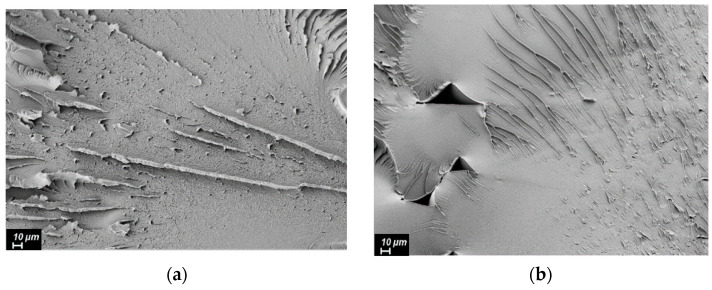

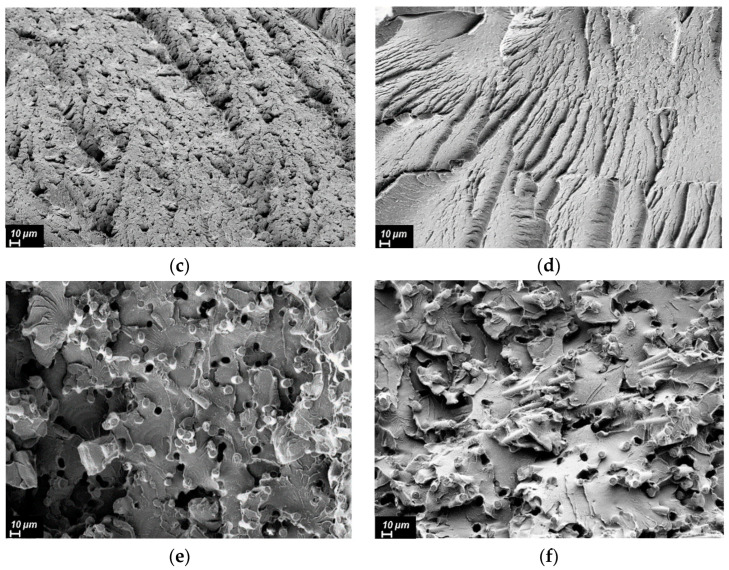
SEM images of the fracture surface of the studied samples (injection molding on the left; FFF printing on the right) from: (**a**,**b**)—pure R-BAPB; (**c**,**d**)—R-BAPB + 1% VGCF; (**e**,**f**)—R-BAPB + 20% CF.

Based on the collected data on the composite’s viscosity, as well as on their thermal and mechanical characteristics, the filaments and, consequently, the samples were obtained by FFF printing from pure R-BAPB, and also filled with 1 wt.% VGCF and 20 wt.% CF. The samples were printed with a raster angle of ±45°, which is the optimal direction for FFF printing to achieve good mechanical properties for most types of tests and can also overlap the pores by changing the direction of the lines. The results of the tensile tests (Figure 7) showed that the strength and Young’s modulus of the printed samples from pure R-BAPB are very close to the parameters of the molded samples (91 and 93 MPa, 2462 and 2179 MPa, respectively). At the same time, the deformation at break in the case of the printed samples does not exceed 10.5%, which is significantly lower than that of the injection-molded samples. This fact is explained by the presence of voids inside the sample caused by layer-by-layer deposition of materials (Figure 8b). FFF samples from R-BAPB filled with 1 wt.% VGCF have a strength and Young’s modulus that are almost identical to the printed samples from pure R-BAPB. However, the deformation at break is almost 4 times higher compared to the unmodified printed R-BAPB sample and this value is 37%. This effect is probably caused by a decrease in porosity and a possible increase in adhesion between layers [41]. The SEM analysis revealed that the voids in the R-BAPB samples with 1 wt.% VGCF become smaller compared to those of pure R-BAPB (Figure 8c,d), and the boundaries between adjacent layers become blurred (Figure 8d). For pure R-BAPB, the fracture surface is sufficiently smooth, which is a typical characteristic of brittle fracture (Figure 8b) [42]. With the introduction of 1 wt.% VGCF, the texture of the fracture surface of the printed sample (Figure 8g) becomes rougher, i.e., plastic destruction takes place. All this suggests that VGCF effectively distorts the crack tip trajectory and increases the complexity of crack propagation [43], which leads to an increased value of deformation at break in the printed R-BAPB sample with 1 wt.% VGCF compared to the pure R-BAPB sample. The samples made of R-BAPB modified with the discrete CF, in turn, have low deformation, but at the same time, their strength increases by almost 50%, and the modulus is 2.5 times higher compared to pure R-BAPB. On the images of the fracture surface, it can be seen how the fibers line up along the direction of laying the polymer thread under 45°, which provides a reinforcing effect (Figure 8e).

For a more detailed study of the internal structure of the samples, their porosity was investigated. The study by the pycnometric method showed that for all samples obtained by injection molding, the porosity barely exceeds 2%, while for the samples obtained by FFF printing, the porosity for pure R-BAPB is ~4% (Table 2), which leads to a decrease in the deformation of the printed sample compared to molding by almost 7 times. The porosity of the sample with 1 wt.% VGCF is only 1.9%, while the deformation and strength properties are comparable to the samples obtained by injection molding, in particular, the printed sample has a high deformation at break of 37%. It can be concluded that the incorporation of VGCF reduces the number of pores in the sample that appear because of the layered deposition of the material, which leads to the fact that the deformation at break increases significantly. For 20 wt.% CF, the porosity of the printed sample is ~5%. In this case, the incorporation of the rigid carbon fibers results in a serious drop in deformation and an increase in Young’s modulus. Summing up, in terms of strength characteristics for FFF-printing, composites based on R-BAPB with discrete carbon fiber exceed materials based on PEEK and Ultem with discrete reinforcing fibers. In turn, for composite materials with carbon nanoparticles, the deformation at break of printed samples exceeds all known analogs.

### 3.4. Investigation of Cytotoxicity of the Obtained Products

Materials that will be in direct contact with living tissue should not have a negative impact on the organism with which they will interact. Exposure to toxic materials can lead to irreversible damage or even the death of cells. Therefore, for use in medicine, any materials are first checked for the level of cytotoxicity through laboratory in vitro tests and analyses. Cells interacting with the test material should not change their normal cycle of functioning, and their proliferation should not be disrupted. In turn, the materials that are planned to be used for implantation should ensure good attachment and growth of cells on their surface.

**Figure 9:**
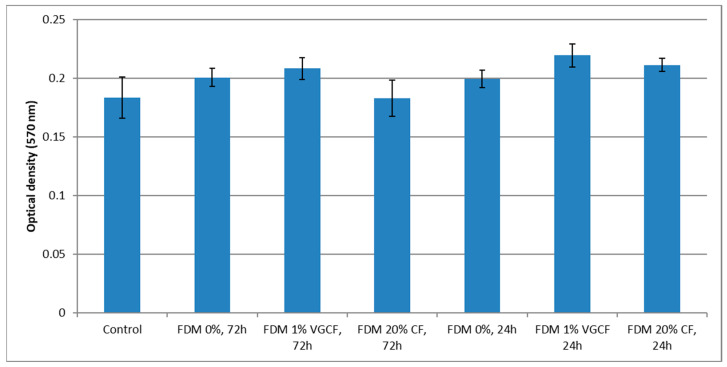
Comparative characteristics of the optical density of formazane solutions obtained from the studied samples.

**Figure 10:**
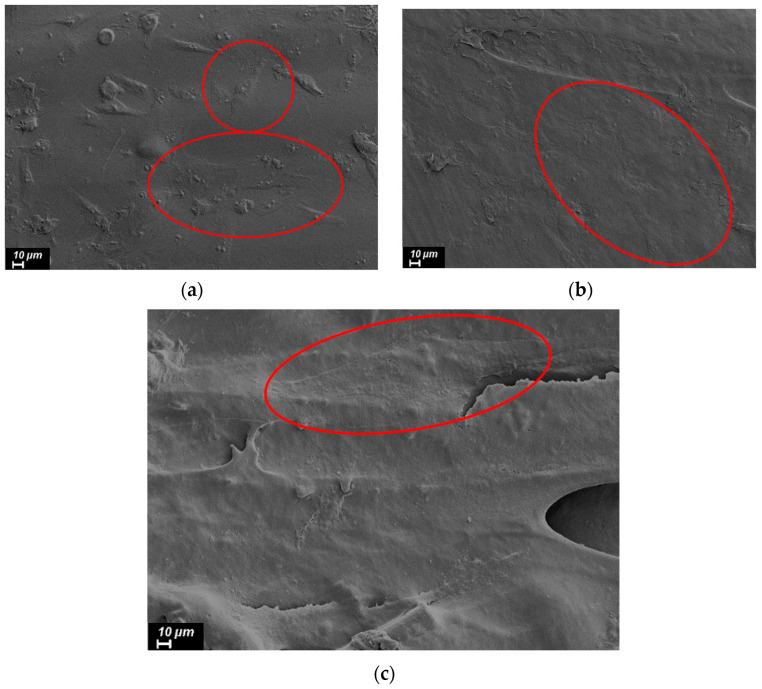
SEM images of cells 1 day after plating on the samples surface prepared by the FFF method from (**a**) pure R-BAPB, (**b**) R-BAPB + 1% VGCF, and (**c**) R-BAPB + 20% CF.

The viability and proliferative activity of cells incubated on the surface of the material was studied using an MTT test. Incubation of cells on the printed samples from pure R-BAPB showed that this material does not have a pronounced cytotoxic effect on human osteosarcoma cell culture. At the same time, when comparing the optical densities of formazane solutions between the levels of proliferative activity of cells incubated on both pure R-BAPB and R-BAPB modified with various fillers, no statistically essential difference was revealed (Figure 9). In the photographs, where cell culture was recorded a day after plating (Figure 10), taken with a scanning electron microscope, it is clear that the cells are well spread out on the surface of the sample. This circumstance indicates that the material does not have a pronounced toxic effect and the surface properties of the samples are favorable for adhesion and proliferation of human osteosarcoma cells.

## 4. Conclusions

In this work, composite materials based on semicrystalline polyimide R-BAPB filled with the carbon nanofibers and micron-sized discrete carbon fibers were obtained.

Based on the study of the melt viscosity of the composites with R-BAPB, and thermal and mechanical properties, the optimal concentration of the carbon nanoparticles in polyimide R-BAPB was determined as 1 wt.%, and for the discrete carbon fibers—20 wt.%.

A study of the printed samples revealed that the introduction of 1 wt.% VGCF reduces the porosity of the printed samples by more than 2 times, which leads to an increase in deformation at break by more than 3 times compared to pure R-BAPB. When 20 wt.% of discrete carbon fibers are added to R-BAPB, there is a sharp increase in the strength of the printed samples to 135 MPa, and the modulus of elasticity to 6.2 GPa.

There was no cytotoxic effect of the polyimide composite materials on the culture of human osteosarcoma cells. Moreover, a good cell adhesion on the surface of the material was observed, indicating the bioinertness of the investigated composites.

Due to the high strength characteristics when incorporating discrete carbon fibers and high deformation when incorporating carbon nanofibers, the developed biocompatible composite materials for FFF can be widely used both in various industrial sectors and in medicine.

## Figures and Tables

**Table 1:** The dependence of the thermal properties of the composites based on R-BAPB on the concentration of various fillers. (*T_g_* is the glass transition temperature, *T_m_* is the melting point, *T_cr_* is the crystallization temperature, *χ* is the degree of crystallinity, *τ_5_* is the temperature at the loss of 5% of the sample mass; the deviation for each value did not exceed 0.5%).

Sample	*T_g_*, °C	*T_m_*, °C	*T_cr_*, °C	*χ*, %	*τ_5_*, °C
R-BAPB	201	326	305	2.7	526
R-BAPB + 0.5% VGCF	201	320	289	3.7	513
R-BAPB + 1% VGCF	200	320	287	13.3	511
R-BAPB + 3% VGCF	201	320	287	16.3	510
R-BAPB + 5% VGCF	200	322	282	23.5	513
R-BAPB + 10% CF	199	320	290	6.5	534
R-BAPB + 20% CF	199	324	289	6.3	535

**Table 2:** Comparison of porosity of the samples from R-BAPB-based composites produced by injection molding (IM) and FFF printing.

Sample	IM	FFF
R-BAPB	0.33 ± 0.07%	3.84 ± 0.13%
R-BAPB + 1% VGCF	1.04 ± 0.05%	1.90 ± 0.09%
R-BAPB + 20% CF	2.13 ± 0.11%	5.27 ± 0.15%

## Data Availability

Not applicable.
